# Multi-Omics Approach to Dissect the Mechanisms of Rexinoid Signaling in Myoblast Differentiation

**DOI:** 10.3389/fphar.2021.746513

**Published:** 2021-09-17

**Authors:** Saadia Khilji, Yuan Li, Jihong Chen, Qiao Li

**Affiliations:** ^1^ Department of Cellular and Molecular Medicine, Faculty of Medicine, University of Ottawa, Ottawa, ON, Canada; ^2^ Department of Pathology and Laboratory Medicine, Faculty of Medicine, University of Ottawa, Ottawa, ON, Canada

**Keywords:** chromatin, gene regulation, histone acetylation, rexinoid, stem cell differentiation

## Abstract

Stem cells represent a key resource in regenerative medicine, however, there is a critical need for pharmacological modulators to promote efficient conversion of stem cells into a myogenic lineage. We have previously shown that bexarotene, an agonist of retinoid X receptor (RXR) approved for cancer therapy, promotes the specification and differentiation of skeletal muscle progenitors. To decipher the molecular regulation of rexinoid signaling in myogenic differentiation, we have integrated RNA-seq transcription profiles with ChIP-seq of H4K8, H3K9, H3K18, H3K27 acetylation, and H3K27 methylation in addition to that of histone acetyl-transferase p300 in rexinoid-promoted myoblast differentiation. Here, we provide details regarding data collection, validation and omics integration analyses to offer strategies for future data application and replication. Our analyses also reveal molecular pathways underlying different patterns of gene expression and p300-associated histone acetylation at distinct chromatin states in rexinoid-enhanced myoblast differentiation. These datasets can be repurposed for future studies to examine the relationship between signaling molecules, chromatin modifiers and histone acetylation in myogenic regulation, providing a framework for discovery and functional characterization of muscle-specific loci.

## Introduction

Epigenetic mechanisms control the patterns of gene expression in stem cell differentiation and play an essential role in regulating the balance between expression patterns for self-renewal of stem cells and activation of lineage-specific genes (Asp et al., 2011; Sincennes et al., 2016; Barreiro and Tajbakhsh, 2017). A thorough understanding of how epigenome dynamics reshape the transcriptional landscape during cell state transitions can facilitate therapy development to manipulate gene expression and promote tissue regeneration. While skeletal muscle has the capacity to regenerate after injury or pathological insult, additional interventions are needed to achieve efficient repair and functional regeneration but are critically lacking (Liu et al., 2018).

Proper formation of skeletal muscle is orchestrated by sequential expression of myogenic regulatory factors (MRFs). Amongst the MRFs, Myf5 and MyoD initiate the transcription of muscle-specific genes, directing cells into the skeletal muscle lineage, whereas myogenin mainly regulates late myoblast differentiation and the fusion of myoblasts into myotubes (Rudnicki and Jaenisch, 1995; Zammit, 2017). We have shown previously that bexarotene, a selective agonist for retinoid X receptor (RXR), promotes the specification and differentiation of the skeletal muscle lineage (Le May et al., 2011; AlSudais et al., 2016). Thereafter, to characterize the nature of RXR signaling in epigenetic landscape and resultant myogenic transcriptome, we performed RNA-seq with condition matching ChIP-seq of five histone modification marks and the histone acetyl-transferase (HAT) p300 in differentiating myoblasts in the absence or presence of bexarotene (Hamed et al., 2017; Khilji et al., 2020). We validate our datasets by recapitulating existing knowledge of myogenic signaling pathways and gain further insights into the impact of RXR signaling on epigenetic modifiers.

Epigenetic mechanisms, including post-translational modification of histones, regulate chromatin structure and accessibility of genes to the transcriptional machinery. Histone modifications may result in disruption of histone-DNA interactions, leading to an open chromatin structure that is accessible to binding of transcriptional machinery or may strengthen histone-DNA interactions to create a tightly packed chromatin structure which favors gene silencing (Wu and Sun, 2006). Based on the type of modification, previous studies have associated patterns of chromatin modifications to distinct regulatory loci, such that H3K9ac, H3K18ac, and H3K27ac are mainly located near transcription start sites (TSS), whereas H4K8ac is associated to promoters and transcribed regions of active genes (Li et al., 2007; Wang et al., 2008; Di Cerbo and Schneider, 2013). Interestingly, H3K4me1 and H3K27ac signify active enhancers and the occurrence of H3K4me1 alone or with H3K27me3 denotes a poised state, whereas accumulation of H3K27me3 is associated with gene silencing (Barski et al., 2007; Creyghton et al., 2010; Rada-Iglesias et al., 2011). In addition to histone marks, enhancers are also characterized by the presence of p300, a transcriptional co-activator involved in many cellular processes including proliferation and differentiation (Ait-Si-Ali et al., 2000, 300; Kung et al., 2000). Knockout of p300 results in severe myogenic abnormalities (Tanaka et al., 1997; Yao et al., 1998), such that derived stem cells are unable to express MyoD or Myf5, underlining a critical role of p300 in myogenic differentiation (Roth et al., 2003). While p300 is widely regarded as a key signature of enhancer activity with H3K18 and H3K27 as its major acetylation substrates (Heintzman et al., 2007; Visel et al., 2009; Jin et al., 2011), we recently reported an increased association of p300 to promoter regions in response to RXR signaling (Khilji et al., 2020).

As global analyses of the combinations of histone marks can be exploited to delineate distinct lineage specific chromatin states (Ernst et al., 2011; Ernst and Kellis, 2012), we have generated and utilized a 14-state chromatin model based upon genome-wide co-occurrence of various epigenetic marks, including those described in this report, to examine the regulation of myogenic enhancers (Hamed et al., 2017). Relatedly, we have performed H4K8ac, H3K9ac, H3K18ac, H3K27ac, H3K27me3 and p300 ChIP-seq using myoblasts differentiated for 24 h in the absence or presence of bexarotene to survey global epigenetic changes and chromatin state dynamics in response to RXR signaling. Our ChIP-seq is paralleled with RNA-seq in matching conditions to support correspondence of genomic landscape with patterns of gene expression. Integration of our OMIC datasets with a myogenic chromatin state model has permitted us to previously characterize an enrichment of loci-specific acetylation of H4K8 and H3K9 at myogenic enhancers by p300 in concert with muscle master regulator MyoD (Khilji et al., 2018). Additionally, we have reported an overlap of late muscle-specific regulator, myogenin, with rexinoid-responsive gene expression where RXR signaling enhances residue-specific histone acetylation at p300 and myogenin co-occupied enhancers (Khilji et al., 2020).

**FIGURE 1:**
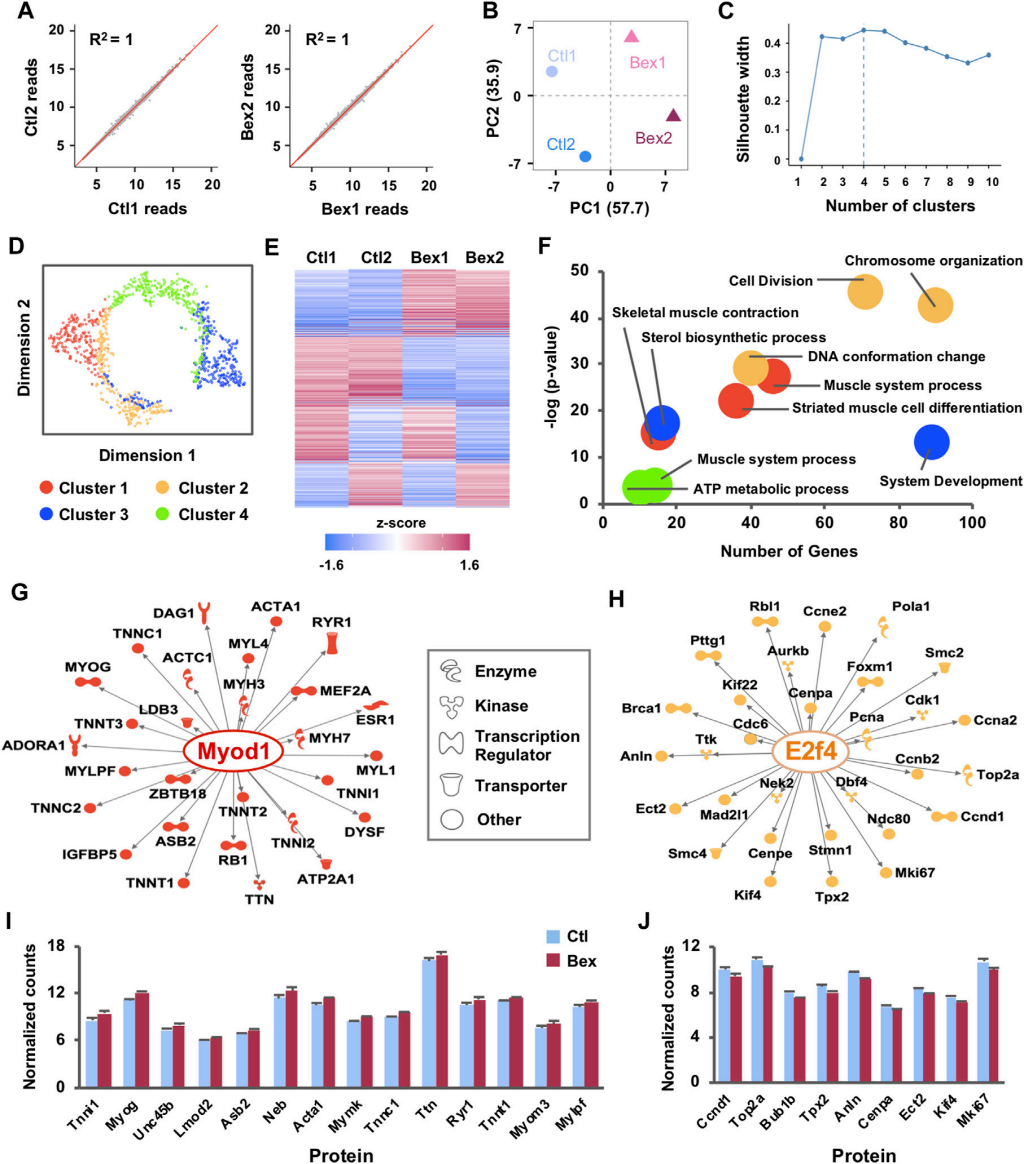
Gene expression clusters reveal distinct gene regulatory networks activated by RXR signaling in myoblast differentiation. **(A)** C2C12 myoblasts were differentiated for 24 h in the absence or presence of bexarotene and subjected to RNA-seq analysis in two replicates (Ctl1 and Ctl2; Bex1 and Bex2). Correlation scatter plots of normalized read counts are shown with linear regression and coefficients of determination (R^2^). **(B)** PCA plot of normalized read counts shows the amount of variance across replicates and conditions. **(C)** The Silhouette method was applied to the top 1,000 most variable genes to determine the optimal number of clusters. The average silhouette width was maximized at k = 4 clusters. **(D)** Dimension reduction algorithm was applied to create a t-SNE map which illustrates the relationships between clusters of co-expressed genes. **(E)** Heat map of k-means clustering on expression profiles normalized by mean center for the 1,000 most variably expressed genes across RNA-seq replicates. **(F)** Gene ontology terms enriched in each cluster are displayed. **(G)** The Regulator Effects tool of the IPA package was employed to identify up-stream regulators of the networks of differentially expressed genes related to muscle development from Cluster I and **(H)** cell cycle related genes from Cluster II. The molecular networks display direct gene interactions only (p-value < 0.01) and the shapes of the nodes reflect the functional class of each gene product. **(I)** Expression patterns of a subset of differentially expressed genes from Cluster I and **(J)** Cluster II, presented as normalized RNA-seq read counts.

**FIGURE 2:**
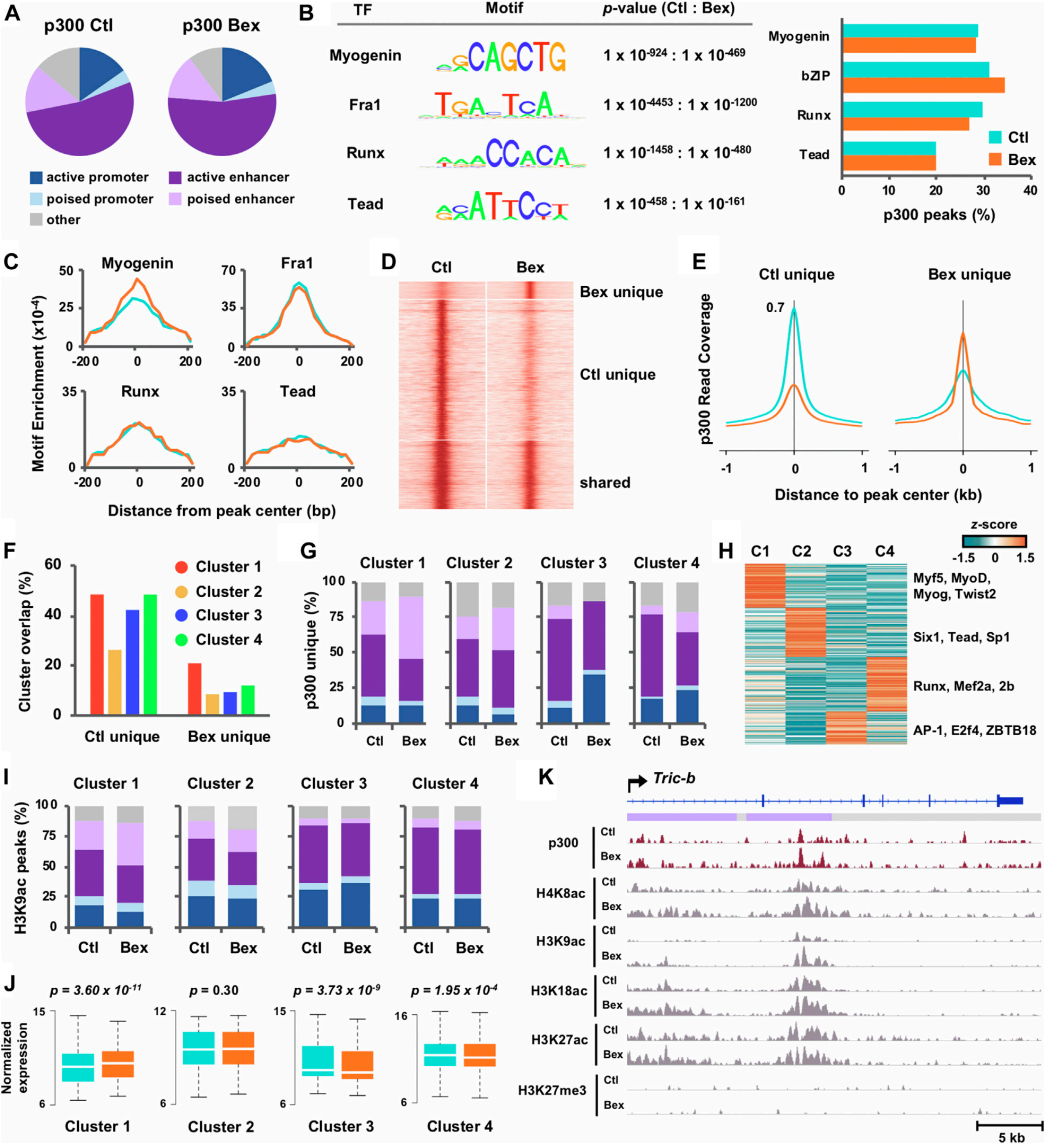
Characterization of p300 loci in relation to chromatin state and histone acetylation in rexinoid enhanced myoblast differentiation. **(A)** Chromatin state distribution of p300 loci in C2C12 myoblasts differentiated for 24 h in the absence or presence of bexarotene (Ctl and Bex). **(B)** The most significant motifs predicted by *de novo* motif discovery of p300 loci are displayed with *p*-values and the percentage of p300 loci associated to each motif are presented to the right. **(C)** Distribution of motif enrichment. **(D)** Signal intensity for p300 was centred around p300 peaks (±1 kb), categorized into sites that were unique to control or bexarotene, and those shared between both conditions. **(E)** Average read density of p300 across p300 loci unique to each condition. **(F)** Gene expression clusters as classified in Panel 1E were associated with p300 loci. The percentage of genes associated to a p300 peak in each cluster is displayed. **(G)** Chromatin state distribution of p300 loci unique to control or to bexarotene and associated to each cluster. **(H)** Motif analysis was performed for p300 loci unique to bexarotene and associated to Clusters I-IV. The z-score distribution of known motifs found through guided analysis and categorized *via* k-means clustering is shown. **(I)** Chromatin state distribution of H3K9ac loci associated to Clusters I-IV. **(J)** The expression levels of ENSEMBL genes associated to p300 loci unique to bexarotene are presented as normalized read counts measured by RNA-seq (paired Wilcoxon rank sum test). **(K)** Genome browser view of p300, and histone modification read density at the Tric-b locus. Blue bars show Refseq gene position and the colors of ChromHMM track below correspond to the chromatin state as designated in panel **(A)**.

Here, we provide technical validation of 16 genomic data sets that profile and integrate global gene expression, histone acetylation, and p300 association with chromatin state dynamics in an effort to obtain an unbiased, genome-wide view of rexinoid-enhanced myoblast differentiation. To facilitate interpretation of this data, we provide detailed information on quality control (Supplementary Figures S1, S2), validation and omics integration analyses (Figures 1, 2). Our goal is to provide insights into rexinoid signaling during myoblast differentiation and create a basis for integrative analyses with future datasets to facilitate the understanding of how the myoblast genome is epigenetically altered in specific signaling pathways or disease states. Furthermore, a comprehensive understanding of the epigenome and transcriptome in myoblasts may provide a framework to identify genetic targets and molecular mechanisms for epigenetic therapies of skeletal muscle-related diseases*.*

## Methods

### RNA-Seq Quality Control

RNA samples from two biological repeats of differentiating myoblasts in the absence or presence of bexarotene were processed by McGill University Genome Quebec Innovation Centre for transcriptome library construction and sequenced using an Illumina HiSeq 2000 sequencer as single-end 50 base pair reads. To assess the quality of reads in Illumina RNA-seq datasets, the mean per sequence quality scores, per base quality scores, and GC content were analyzed using the FastQC program (Andrews, 2010). The quality scores across reads for all data sets fell within a high confidence range with base quality scores above 30 and the average GC content across reads represents an overall normal distribution of a standard random library (Supplementary Figures S1A–C). In addition, the average duplication rate across all four samples was approximately 40%, below the normal duplication rate cut-off of 50%, indicating sufficient starting material and library complexity (Supplementary Figure S1D). Subsequently, Salmon (Patro et al., 2017) was used to perform mapping based estimation of transcript abundance while the gene body coverage tool from the RNA-seq Quality Control package (Wang et al., 2012) was used to assess for uniformity of read coverage across the gene body for indications of any 5'/3′ bias in sequencing. As shown in Supplementary Figure S1E, approximately equal percentage of reads mapped near the 5′ and 3′ ends as well as a similar coverage across samples, indicated relatively even coverage of reads across the gene body. Moreover, Supplementary Figures S1F, shows the exonic rate was similar across all samples.

### ChIP-Seq Quality Control

Since cellular transcription patterns are impacted by epigenetic modifications, we analyzed ChIP-seq of histone marks including H4K8ac, H3K9ac, H3K18ac, H3K27ac, and H3K27me3. Genomic localization of p300 was also examined as it is a critical HAT for myogenic differentiation (Puri et al., 1997; Polesskaya et al., 2001; Roth et al., 2003). Similar to the RNA-seq datasets, ChIP-seq library preparation and sequencing was performed by the McGill University Genome Quebec Innovation Centre with Illumina HiSeq 2000 as single-end 50 bp reads. As shown in Supplementary Figure S2A, the average base quality scores for all data sets were higher than a threshold Phred quality score of 30 and also displayed an overall normal distribution for the average GC content of reads (Supplementary Figure S2B). Next, sequence reads were aligned to the mm9 reference genome using Bowtie2 (Langmead and Salzberg, 2012) with subsequent removal of duplicate reads by Picard tools (Picard Toolkit, 2019). Examination of genome coverage and ChIP enrichment simultaneously *via* fingerprint plot analysis displayed intersection of the x-axis at approximately 0.1 by the fingerprint plot traces of all samples indicating an absence of read coverage across 10% of the genome (Supplementary Figure 2C). Given that the rightward deflection of a trace indicates the extent of ChIP enrichment, all datasets reflect ChIP enrichment in comparison to input. Lastly, we assessed the relationship of all ChIP-seq datasets in relation to one another by clustering histone acetylation marks into their respective biological groups for chromatin modification and condition (Supplementary Figure S2D). The PCA plot revealed close clustering of H4K8ac, H3K18ac and H3K27ac, all of which were distinct from H3K9ac. The division of H3K9ac from other histone marks reflects a recent report where H3K9ac was specifically enriched at p300-associated myogenic enhancers and appears to be a signature of rexinoid-responsive gene expression (Khilji et al., 2020). Additionally, p300 displayed separation from histone acetylation marks along the first principal component, that explains 51.5% of the variance, suggesting distinct global localization of p300, as expected of a histone acetyltransferase which affects a large array of cellular processes (Vo and Goodman, 2001).

## Experimental Design

Here, we examined the impact of rexinoid signaling on myogenic gene expression and epigenetic landscape on a genomic scale using C2C12 myoblasts as a model of skeletal myoblast differentiation. This cell type is a model of choice for many genome-wide studies not only due to the high correlation between the transcription profiles of primary and C2C12 myoblasts (Blais et al., 2005; Cao et al., 2010), but also because C2C12 cells are less prone to spontaneous differentiation than primary myoblasts, and thus represent a more homogenous population for large-scale studies (Burattini et al., 2004; Asp et al., 2011). In our study, myoblasts were differentiated for 24 h in the absence or presence of bexarotene to capture early transcriptional dynamics as thousands of genes are differentially expressed within 24 h of differentiation initiation (Trapnell et al., 2010; Doynova et al., 2017; Hamed et al., 2017).

Furthermore, since histone modifications are known to be rather dynamic, an earlier time point warrants the capture of epigenetic alterations in relation to transcriptional modifiers. We thus performed ChIP-seq for H4K8ac, H3K9ac, H3K18ac, H3K27ac, H3K27me3, and p300 in matching conditions of RNA-seq to seize epigenetic changes of common promoter and enhancer marks. Analysis of our dataset in the context of other signaling pathways or transcription factor association will facilitate new biological insights into the chromatin dynamics of myogenic differentiation.

## Validation

In addition to detecting novel transcripts, a principal benefit of RNA-sequencing has been replicability between samples. To test this parameter, gene level counts were obtained *via* the tximport package (Soneson et al., 2016) which estimates counts and transcript lengths for downstream gene-level analysis followed by use of integrated Differential Expression and Pathway (iDEP) (Ge et al., 2018) to filter out genes with less than 0.5 counts per million (CPM) in all four samples. Of the 30,099 Ensembl gene IDs, 14,484 genes passed the filter and were processed *via* a regularized log (rlog) transformation which minimizes differences between samples with small counts and normalizes with respect to library size (Ge et al., 2018). Following the processing, we measured similarity between RNA-seq repeats by pairwise scatter plots which displayed a high degree of replicability between samples (Figure 1A). Likewise, the relationship of rlog-transformed read counts visualized with PCA revealed a clear difference between the bexarotene and the control samples, along the first principal component that explains 57.7% of the variance (Figure 1B). Integration of these results showed reliable biological duplication, indicating that the data obtained in this study could be used for subsequent analyses.

To guide the validation and functional characterization of early myogenic gene expression in response to rexinoid signaling, we used k-means clustering as part of the iDEP software (Ge et al., 2018) to divide the top 1,000 most variable genes into distinctive groups (Figures 1C–E). First, we used the silhouette width method which is an estimate of the average distance between clusters to guide the selection of optimal k clusters. We found that k = 4 provides an optimal reduction without introducing large overlaps amongst clusters (Figures 1C,D). Clusters I-IV had 281, 184, 301, and 234 genes, respectively. Visualization of k-means clustering presented noticeably different expression patterns amongst the clusters where two of the four clusters shared similar expression profiles across replicates (Figure 1E). Cluster I contained genes with increasing expression profiles in bexarotene treated myoblasts compared to Cluster II in which genes were more abundantly expressed in control myoblasts (Figure 1E). Clusters III and IV displayed less homogeneity across replicates as gene expression in one of the control replicates matched more closely to a treated replicate than to the second control and vice versa (Figure 1E). To understand this functional discrepancy between clusters, we used iDEP (Ge et al., 2018) to perform gene ontology analysis to identify significant signaling pathways enriched in each cluster (Figure 1F). Cluster I genes, which were upregulated by bexarotene, were distinctly associated with processes of skeletal muscle development whereas Cluster II genes, downregulated by bexarotene, were associated with the cell cycle and accompanying DNA conformational changes (Figure 1F). Once again, Clusters III and IV displayed greater heterogeneity with association to sterol synthesis and ATP metabolism but notably, with a much lower level of significance (Figure 1F). Due to the agreement across replicates for Cluster I and II as well as an increased significant association with gene ontology enrichment, we focused our remaining analyses on Clusters I and II.

To assess the transcriptional regulation network of the most highly expressed genes in Clusters 1 and II, we utilized upstream regulator analysis as part of Qiagen’s Ingenuity Pathway Analysis (IPA) software (Figures 1G,H). Based on annotated expression of downstream genes, IPA identified MyoD as a prominent upstream regulator (*p* = 2.74 × 10^–32^) of key muscle-specific proteins in Cluster I including myogenin, and the troponin and myosin families which all displayed increased expression in response to bexarotene (Figures 1G,I). In contrast, differential gene expression in Cluster II predicted E2F4 as a main upstream regulator (*p* = 3.00 × 10^–49^). E2F4 is a critical molecule in the RB/E2F pathway and is known to play an important role in control of the cell cycle and action of tumor suppressor proteins (Puri et al., 1998; DeGregori and Johnson, 2006; Chen et al., 2009). As a transcriptional regulator in rexinoid signalling, E2F4 led to reduced levels of Cyclin D1 (Ccnd1) and other cell cycle genes including Top2a, Anln, and Mki67 (Figures 1H,J). Taken together, the overall expression signature of rexinoid signaling in myoblast differentiation indeed reflects activation of pathways related to skeletal muscle development and a synchronized decrease in cell cycle signaling.

## Omics Integration

Following the analyses of rexinoid-responsive myogenic gene expression, we next sought to provide further evidence of biological validity, *via* integration of RNA-seq and ChIP-seq datasets, to depict epigenetic mechanisms of early myogenic gene transcription in rexinoid signaling. We utilized our chromatin state model to characterize p300 localization in myoblasts differentiated for 24 h in the absence or presence of bexarotene (Figure 2A). Similar to previous reports (Heintzman et al., 2007; Visel et al., 2009), approximately two-thirds of p300 peaks in control myblasts were associated to enhancers, which remained unchanged for p300 peaks in response to bexarotene despite a moderate increase in p300 association to promoter regions. Next, we utilized the *de novo* motif discovery tool as part of the Hypergeometric Optimization of Motif EnRichment (HOMER) software (Heinz et al., 2010) with input sequences of 200 base pairs surrounding the peak center, to search for enriched sequence motifs at p300 loci in response to bexarotene (Figure 2B). Motifs matching consensus binding sites for myogenin, bZIP, Runx, and Tead transcription factors were enriched among p300 loci with little change in the percentage of target loci associated to each motif in both conditions (Figure 2B). Interestingly, although p300 association to E-box did not change in response to RXR signaling, it was the only motif for which loci-specific enrichment increased over 1.5-fold, suggesting that RXR signaling generally directs an increased recruitment of p300 by E-box binding proteins to each locus, rather than to an increased number of loci (Figure 2C).

Through categorizing the events of p300 binding, we also observed a greater increase in peak signal intensity at p300 loci unique to control when compared to p300 loci unique to bexarotene, similar to loci shared between both conditions (Figures 2D,E). These results suggest that RXR signaling promotes an increase in p300 enrichment only at a discrete set of loci. To explore the functional characterization of these loci, we integrated gene expression of RNA-seq Clusters I-IV (Figure 1E) with p300 loci unique to control and to bexarotene. As seen in Figure 2F, genes in Clusters I, III, and IV were approximately equally associated with p300 loci unique to control whereas p300 loci unique to bexarotene displayed the greatest association to Cluster I genes. Interestingly, the association of bexarone unique p300 loci to poised enhancers increased from 25 to 44% in response to bexarotene, while p300 association to other chromatin states decreased or remained unchanged in Cluster I (Figure 2G). The association of bexarotene unique p300 loci with poised enhancers increased in Cluster II and IV as well but to a smaller degree. In contrast, p300 loci in Cluster III exhibited a 3-fold increase in association to promoter regions from 12 to 36% following bexarotene treatment, with almost no association to poised enhancers (Figure 2G). These results suggest that RXR signaling strongly stimulates p300 association to poised enhancers in the regulation of myogenic genes within Cluster I, cell cycle genes in Cluster II and an increase to promoter regions for the regulation sterol biosynthetic and metabolic processes in Cluster III (Figure 2G).

To examine protein interactions of p300 stimulated by RXR signaling and the links to each cluster of genes, we performed k-means clustering on the enrichment values of known motifs (z-scores) associated with p300 loci unique to bexarotene. The k-means machine learning algorithm partitioned motifs into a set of four groups such that motifs within the same cluster displayed the most similar enrichment in each condition. As seen in Figure 2H, transcription factors specific to myogenesis including Myf5, MyoD, and myogenin were preferentially found in Cluster I, while Six1, Tead, and Sp1 were enriched in Cluster II (Figure 2H). On the other hand, p300 loci associated to Cluster III which were increasingly associated to promoters in response to bexarotene, were enriched for promoter factors including E2F4 and ZBTB18 (Figures 2G,H). Next, as we have previously reported an increase in residue-specific histone acetylation of H3K9 at enhancers co-occupied by p300 and myogenin in RXR signalling (Khilji et al., 2020), we examined further the correlation of chromatin state in relation to H3K9 acetylation. As shown in Figure 2I, the chromatin state association of H3K9ac loci is approximately similar in control and bexarotene treated myoblasts, bar an increased association to poised enhancers in Cluster I and a slightly smaller increase in Cluster II. An increase in distribution to poised enhancers of H3K9ac loci related to Cluster I genes in RXR signalling was parallel to an increase of p300 association to poised enhancers as well (Figures 2G,I). Poised enhancers, established in lineage progenitors, play a determinant role in the control of genes required for stem cell differentiation and are marked by distinctive histone acetylation and HAT recruitment including p300 (Heinz et al., 2010). Thus, an increased association of H3K9ac at poised enhancers may reflect the activation of discrete lineage-specific gene programs by RXR signaling.

As the p300 peaks in Cluster I and II exhibited an increasing association with poised enhancers (Figure 2G), we examined the corresponding gene expression as determined by RNA-seq in matching conditions (Figure 2J). Interestingly, Cluster I genes associated to p300 loci unique to bexarotene displayed a significant increase in expression, which was lacking in the other three clusters, where Cluster III and IV genes displayed a significant decrease in expression instead (Figure 2J). Moreover, the enrichment of p300 in response to rexinoid signaling at the putative regulatory locus of Tric-b, a cation channel regulating intracellular calcium release (LmYazawa et al., 2007), represents an individual example of global patterns where p300 occupancy at a Cluster I gene locus correlates with an increase in histone acetylation (Figure 2K).

## Conclusion

Taken together, the integration of p300 ChIP-seq with chromatin state classification and chromatin modification coverage can be used to delineate the distinct functions of HATs in the regulation of myogenic expression in specific biological contexts such as rexinoid responsive gene expression. Additionally, the model of rexinoid-enhanced myogenesis offers a system to identify new genetic targets and molecular interactions which may have pharmacological significance related specifically to muscle-related disease. This report represents biologically valid and high-quality next generation sequencing data that will be useful for future studies examining the role of novel and known myogenic regulators including p300, histone acetylation and their relation to chromatin state dynamics in early myogenic differentiation.

## Data Availability

The datasets presented in this study can be found in online repositories. The names of the repository/repositories and accession number(s) can be found below: https://www.ncbi.nlm.nih.gov/geo/, GSE94561 https://www.ncbi.nlm.nih.gov/geo/, GSE139942.
