# Supplementary figures and images for: Multi-Omics Approach to Dissect the Mechanisms of Rexinoid Signaling in Myoblast Differentiation
Source: Front Pharmacol. 2021 Sep 17;12:746513. doi: 10.3389/fphar.2021.746513 (PMC8484533; doi:10.3389/fphar.2021.746513)

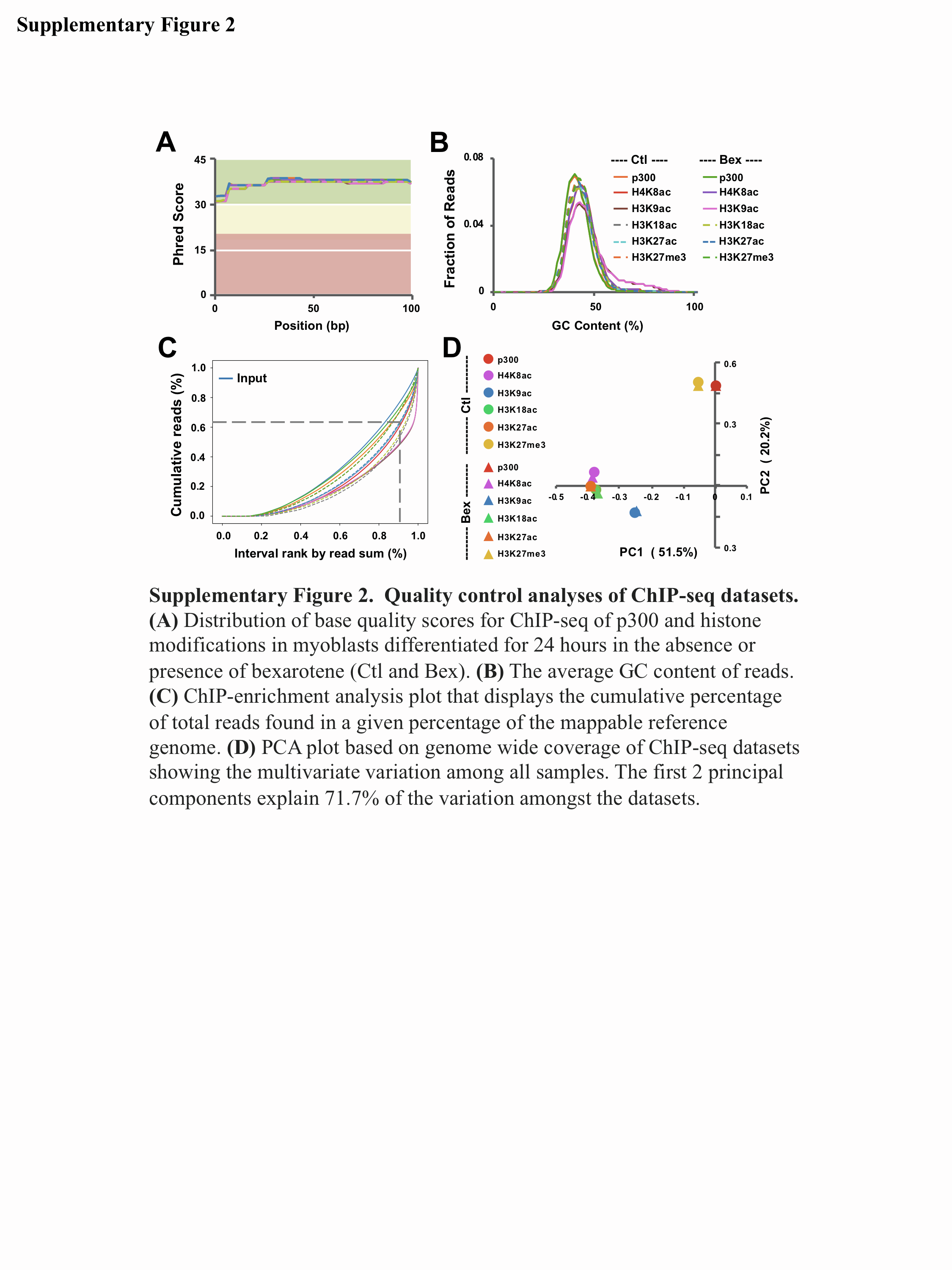

Supplement: Supplementary file 1 [file Image2.tif]

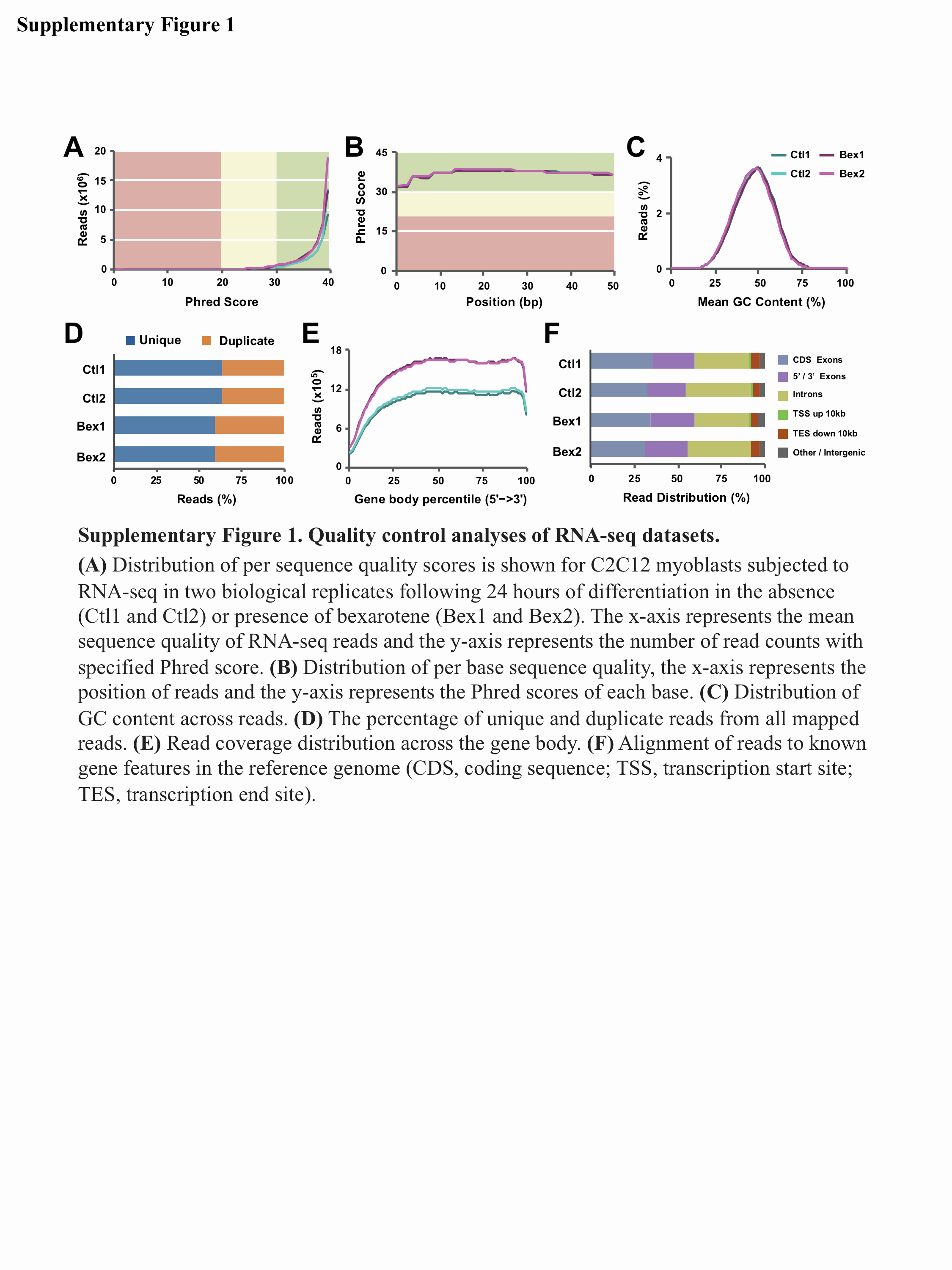

Supplement: Supplementary file 2 [file Image1.tif]
